# Nanobody-enhanced chimeric antigen receptor T-cell therapy: overcoming barriers in solid tumors with VHH and VNAR-based constructs

**DOI:** 10.1186/s40364-025-00755-5

**Published:** 2025-03-11

**Authors:** Shasha Guo, Xiaozhi Xi

**Affiliations:** 1 Shandong Women’s University, Jinan, 250300 People’s Republic of China; 2 Department of Otolaryngology-Head and Neck Surgery, Shandong Provincial ENT Hospital, Shandong University, Jinan, 250022 People’s Republic of China; 3 Oncology Department, Shandong Second Provincial General Hospital, Jinan, 250023 People’s Republic of China; 4 Key Laboratory of Marine Drugs, School of Medicine and Pharmacy, Ministry of Education, Ocean University of China, Qingdao, 266003 People’s Republic of China

**Keywords:** CAR-T, Single domain antibody, VHH, VNAR, Solid tumor

## Abstract

CAR-T cells are genetically modified T lymphocytes that express chimeric antigen receptors (CAR) on their surfaces. These receptors enable T lymphocytes to recognize specific antigens on target cells, triggering a response that leads to targeted cytotoxicity. While CAR-T therapy has effectively treated various blood cancers, it faces significant challenges in addressing solid tumors. These challenges include identifying precise tumor antigens, overcoming antigen evasion, and enhancing the function of CAR-T cells within the tumor microenvironment. Single domain antibody, versatile tools with low immunogenicity, high stability, and strong affinity, show promise for improving the efficacy of CAR-T cells against solid tumors. By addressing these challenges, single domain antibody has the potential to overcome the limitations associated with ScFv antibody-based CAR-T therapies. This review highlights the benefits of utilizing single domain antibody in CAR-T therapy, particularly in targeting tumor antigens, and explores development strategies that could advance the field.

## Graphical Abstract

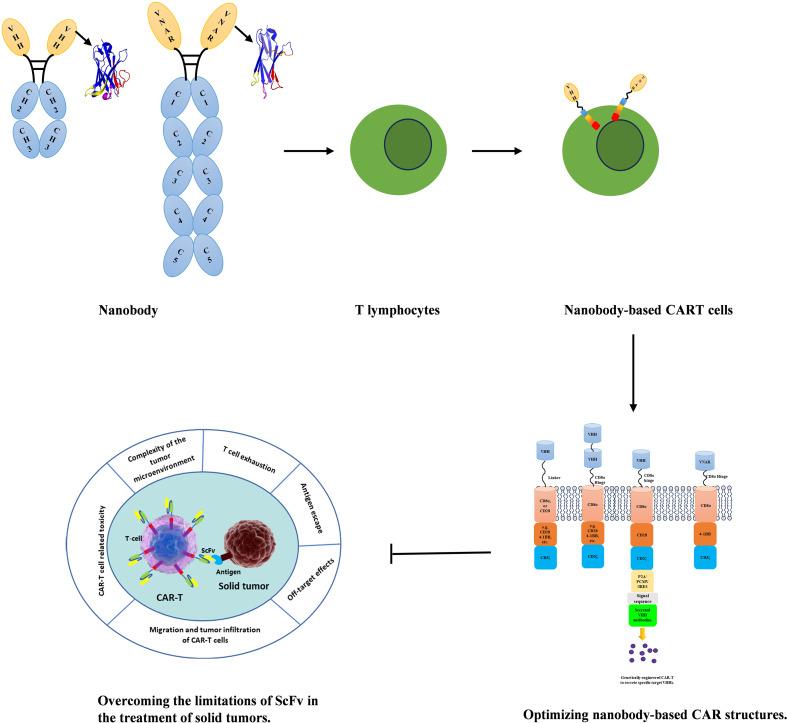

## Introduction

Cell therapy has emerged as a transformative approach in cancer treatment, with chimeric antigen receptor (CAR) T cells standing out as a particularly potent modality [1, 2]. CAR-T cells are genetically engineered T lymphocytes that express synthetic CAR molecules on their surface, enabling them to recognize specific target antigens and initiate cytotoxic responses independently of the major histocompatibility complex (MHC) [3, 4]. This innovative therapy has demonstrated remarkable success in hematological malignancies, such as leukemias and lymphomas, with FDA-approved therapies like Tisagenlecleucel and Axicabtagene ciloleucel targeting CD19, and Idecabtagene Vicleucel and Ciltacabtagene Autoleucel targeting B cell maturation antigen (BCMA) [5, 6]. CAR-T cells are particularly effective in treating hematological malignancies because of the consistent presence of target antigens such as CD19 and BCMA, which allow for precise targeting of cancer cells [7–10]. Single-chain variable fragments (ScFv) are commonly utilized as the antigen recognition region in CAR constructs, comprising a variable heavy chain (VH) and a variable light chain (VL) connected by a flexible linker (Gly4Ser)3 [11–14]. The folding of artificially designed ScFv can affect the specificity and affinity of CAR towards its target antigen, ultimately impacting the effectiveness of CAR-T therapy [15]. With the development of biotechnology, single domain antibody have gradually entered the application of CAR-T therapy with their unique structures and functions, and can provide potential substitutes as the antigen recognition domain of CAR [16, 17]. A notable advancement in CAR-T therapy is the incorporation of single-domain antibodies (sdAbs), such as VHH from camelids, as antigen recognition domains. The most prominent example is Ciltacabtagene Autoleucel (Carvykti), a VHH-based CAR-T therapy targeting BCMA, which received FDA approval in 2022 for the treatment of relapsed or refractory multiple myeloma [18]. Carvykti utilizes two VHH domains to enhance antigen binding specificity and efficacy, demonstrating the potential of sdAbs in improving CAR-T cell performance [18]. However, the application of CAR-T therapy in solid tumors has been significantly limited due to unique challenges, including the immunosuppressive tumor microenvironment (TME), tumor heterogeneity, and the lack of suitable target antigens [19–21]. Traditional single-chain variable fragment (ScFv)-based CAR designs, while effective in hematological malignancies, often fail to address these barriers in solid tumors. This has spurred the exploration of alternative antigen recognition domains, such as VHH and VNAR from sharks. These sdAbs offer distinct advantages, including small size, high stability, and the ability to bind cryptic epitopes, making them promising candidates for CAR-T engineering [22].

This review focuses on the structural and functional advantages of sdAbs over traditional ScFv-based CARs, particularly in the context of solid tumors. By leveraging the unique properties of VHH and VNAR, this study aim to overcome the limitations of conventional therapy.

### The development history of CAR-T cell therapy

**Fig. 1:**
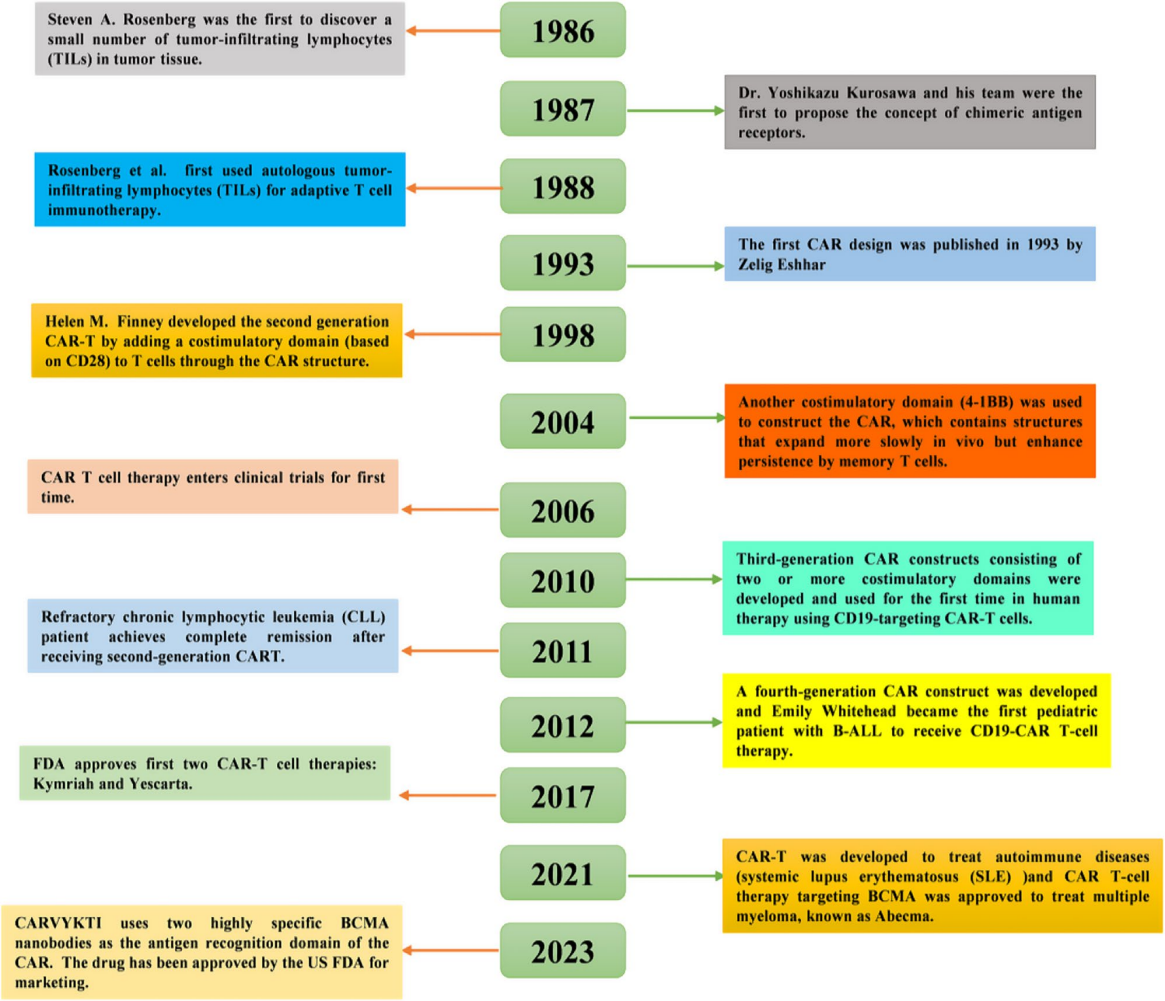
Timeline of the development of CAR-T cell therapy

The evolution of CAR-T cell therapy began in the late 1980s with the discovery of tumor-infiltrating lymphocytes (TILs) and the concept of chimeric T cell receptors (Fig.  1), progressing through key milestones to become a clinically validated treatment [23, 24]. The first-generation CAR-T cells, developed in the early 1990s, showed feasibility but were hindered by poor persistence and efficacy [25]. The incorporation of costimulatory domains, such as CD28 or 4-1BB [26–29], in second-generation CAR-T cells marked a major advancement, enhancing T cell proliferation and anti-tumor activity [30–32]. Further refinements led to third-generation CAR-T cells, which combined multiple costimulatory molecules to improve functionality [33–35]. Clinical breakthroughs in the 2010s, including the successful treatment of relapsed or refractory B-cell malignancies, paved the way for FDA approvals of CAR-T therapies like Kymriah and Yescarta [36–38]. In 2022, Carvykti, a VHH-based CAR-T therapy for relapsed multiple myeloma, was approved, showcasing the potential of single-domain antibodies to enhance CAR-T cell specificity and efficacy [18].

More recently, the integration of single-domain antibodies, such as VHH and VNAR, into CAR designs has opened new avenues for targeting solid tumors and overcoming the limitations of traditional ScFv-based CARs [39–41]. These advancements, coupled with innovations in CAR design, manufacturing processes, and safety measures, continue to propel CAR-T cell therapy forward [42, 43]. This progression from foundational research to clinical practice underscores CAR-T’s transformative potential in cancer treatment.

## Challenges of CAR-T cell therapy in solid tumors: leveraging VHH and VNAR single-domain antibodies for enhanced efficacy

CAR-T therapy is currently a topic of great interest, with 10 approved products for marketing worldwide (see Table 1). These marketed CAR-T products predominantly target CD19 and BCMA, both of which are expressed in B cells, making them suitable for treating B cell-related malignancies and myeloma due to their limited immune evasion, lack of cross-reactivity in multiple tissues, and stable expression [44, 45]. Notably, CAR-T therapy targeting CD19 for myeloma has shown a treatment success rate as high as 90% [4]. While the success of CD19 and BCMA targets is significant, especially in B cell-related malignancies, relying solely on these two targets poses limitations. Currently, CAR-T therapy is limited to B cell-related hematological malignancies and is not effective against other cancer types like breast cancer or lung cancer. This limited scope of treatment hinders the potential efficacy in other cancer types, emphasizing the need for developing new therapeutic targets and strategies to further advance CAR-T therapy.

**Table 1:** Globally approved and marketed CAR-T cell therapy

Product name	Antigen	Indication	Sponsor	Time-to-market	Complete remission rate	Ref
Kymriah	CD19	Precursor B-cell acute lymphoblastic leukaemia that is refractory or presents with two or more relapses	Novartis Switzerland	2017	90%	[4]
Yescarta	CD19	Adult patients with relapsed or refractory large B-cell lymphoma after two or more systemic therapies	Gilead Sciences, USA	2017	51%	[46]
Tecartus	CD19	Adult patients with relapsed or refractory lymphoma of the condyle (MCL)	Gilead Sciences, USA	2020	67	[47]
Breyanzi	CD19	For the treatment of adult patients with relapsed or refractory large B-cell lymphoma after two or more lines of systemic therapy	BMS(Juno), USA	2021	54	[48]
Abecma	BCMA	Adults with recurrent or refractory multiple myeloma after four or more prior therapies, including immunomodulators, proteasome inhibitors and anti-CD38 monoclonal antibodies	BMS(Celgene), USA	2021	28%	[49]
Talicabtagene autoleucel	CD19	Adult patients with relapsed or refractory large B-cell lymphoma (r/r LBCL) after prior second-line or more systemic therapy	ImmunoACT Ltd Indian	2021	54%	[50]
Relmacabtagene autoleucel	CD19	Adult relapsed or refractory large B-cell patient lymphoma (r/r LBCL) after two or more lines of systemic therapy	JW Therapeutics (Shanghai) Co., Ltd. China	2021	52%	[51]
CARVYKTI	BCMA	Adult relapsed/refractory multiple myeloma (r/r MM)	Nanjing Legend Biotech /Xian Janssen Pharmaceutical Ltd. China	2022	78%	[18]
Equecabtagene Autoleucel	BCMA	Adult patients with relapsed or refractory multiple myeloma (MM) who have progressed (with at least one proteasome inhibitor and immunomodulator) after at least 3 lines of prior therapy	Nanjing IASO Biotechnology Co./ Innovent Biopharmaceutical (Suzhou) Co. China	2023	79%	[52]
CNCT19	CD19	Relapsed or refractory B-cell acute lymphoblastic leukaemia (r/r B-ALL) in adults	Juventas Cell Therapy Ltd. China	2023	51%	[53]

**Fig. 2:**
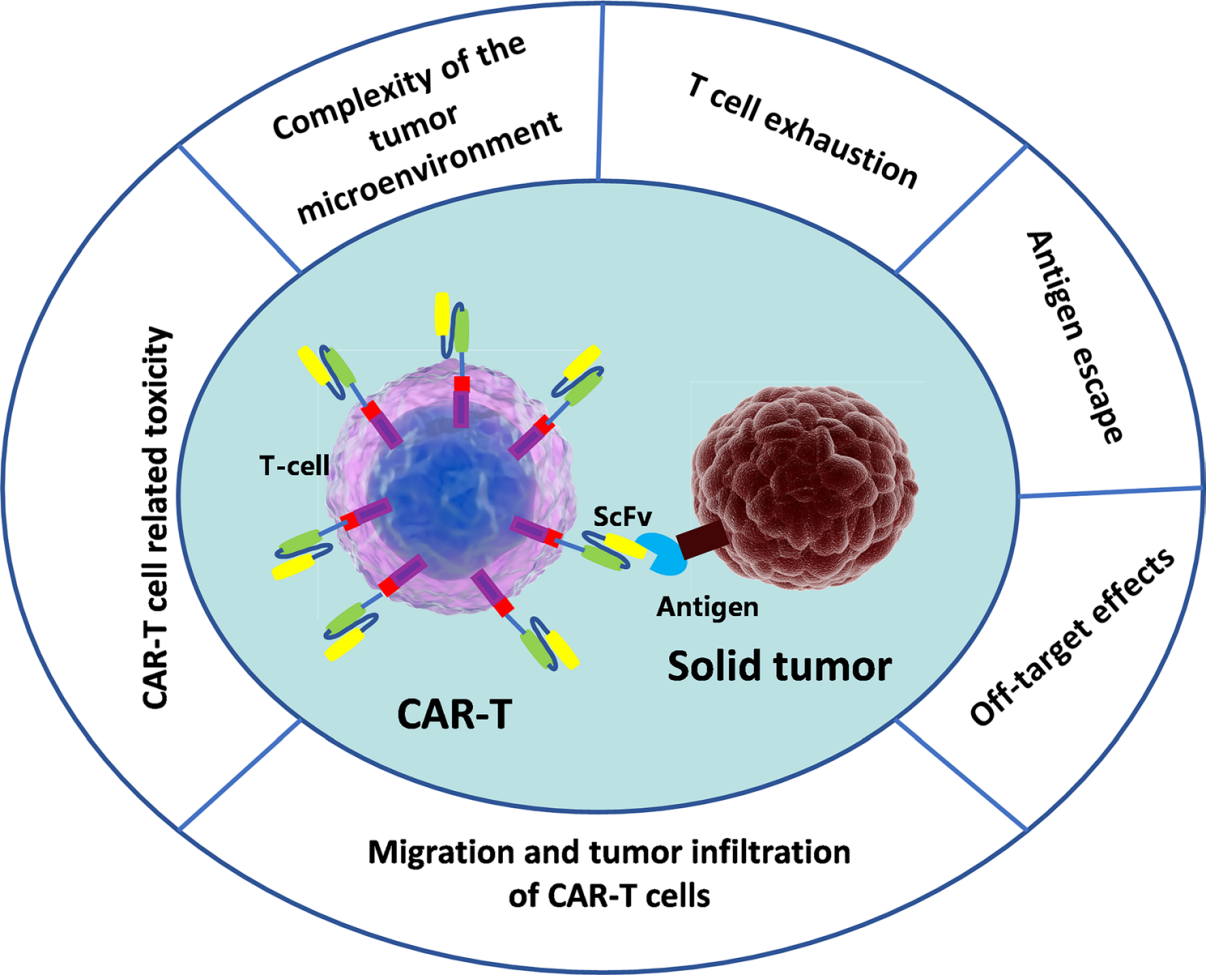
Limitations of CAR-T for the treatment of solid tumors.The picture summarizes the limiting factors of CAR-T cells in the treatment of solid tumors

Compared to hematological tumors, solid tumors have immunosuppressive tumor microenvironment (TME), limited target antigen selection, low T cell infiltration rate, and the characteristics of tumor heterogeneity (Fig. 2). Therefore, the application of CAR-T in solid tumors is not optimistic [54, 55].

### The impact of the immune microenvironment on CAR-T

The efficacy of CAR-T therapy in hematological malignancies is closely linked to the tumor microenvironment (TME). Cancer cells in hematological malignancies circulate in the blood and bone marrow, and reinfused CAR-T cells directly participate in this process, enabling them to localize and target tumor cells [8]. In contrast, the TME of solid tumors presents significant challenges, characterized by features such as low pH, hypoxia, high permeability, and immunosuppression, which impair T-cell survival and immune function [56]. Immunosuppressive cells, including myeloid-derived suppressor cells (MDSCs) [57], tumor-associated macrophages (TAMs) [58], and regulatory T cells (Tregs) [59], are prevalent in solid tumors and secrete cytokines, growth factors, and chemokines, contributing to an immunosuppressive environment. Moreover, the interaction between tumor cells and immune cells fosters a further immunosuppressive microenvironment (Fig. 2) [60]. Solid tumors are also marked by an abundance of tumor-associated fibroblasts and extracellular matrix components, which form physical barriers that limit CAR-T cell access to the tumor site [54, 55]. These tumors may also inhibit chemokine secretion and reduce the expression of relevant receptors on CAR-T cells, preventing effective tumor homing. As a result, only a small fraction of CAR-T cells that manage to infiltrate the tumor site can reach sufficient numbers to achieve therapeutic efficacy [54, 55]. Consequently, CAR-T therapy’s effectiveness in solid tumors has been limited.

However, recent advancements, particularly the use of single domain antibodies in CAR-T cell therapy, offer new hope for overcoming these challenges. Single domain antibodies can be engineered to enhance CAR designs, enabling improved penetration of solid tumors and increased specificity for tumor-associated antigens. These innovations hold promise for improving CAR-T cell therapy’s effectiveness in solid tumors, potentially overcoming the barriers posed by the TME.

### T cell depletion limits CAR-T therapy

One of the major challenges in using CAR-T cells to treat solid tumors is T-cell exhaustion. Solid tumors often feature a dense extracellular matrix and exhibit inhibitory checkpoint signaling, both of which impair the immune response. For example, regulatory T cells and myeloid-derived suppressor cells not only induce exhaustion in CAR-T cells but also suppress the activity of endogenous T cells infiltrating the tumor [61]. As CAR-T cells penetrate the tumor, they can become exhausted, losing their effector functions. Exhausted CAR-T cells fail to secrete IFN-γ, cease proliferating, and show diminished cytotoxic activity [62]. Additionally, mitochondrial dysfunction in these cells further weakens their metabolic reserves, and the upregulation of inhibitory checkpoint receptors such as PD-1, TIM-3, and CTLA-4 contributes to immune escape [54, 55]. Consequently, overcoming T-cell exhaustion through innovations in CAR design is essential for improving the therapeutic potential of CAR-T therapy in solid tumors.

Recent advances in the use of single domain antibodies offer promising strategies for addressing these challenges. By incorporating single domain antibodies into CAR constructs, it is possible to improve tumor targeting, enhance CAR-T cell persistence, and potentially mitigate exhaustion. These innovative CAR designs could offer new hope for solid tumor therapies by overcoming the T-cell exhaustion barrier and enhancing immune efficacy.

### Antigen accessibility in solid tumors

The accessibility of antigens and resistance to CAR-T therapy are significant challenges in cell therapy [63]. In hematological tumors, resistance often arises from the loss of target antigen expression, a phenomenon known as antigen escape [64]. Solid tumors present a greater challenge due to antigenic heterogeneity, where tumor cells can exhibit diverse subpopulations within a single patient or across patients. CAR-T cells are effective only against tumor cells that express the target antigen, and non-targeting cells can proliferate and metastasize, leading to the emergence of dominant subpopulations that are resistant to therapy [65, 66]. To address this, the development of tumor-specific neoantigens for solid tumors is crucial to expand the applicability of CAR-T therapy.

Solid tumors typically contain few tumor-specific antigens (TSAs), and most of the highly expressed antigens are tumor-associated antigens (TAAs). However, TAAs are also found in normal tissues, leading to off-target effects that can reduce the efficacy of CAR-T therapy and cause potential toxicity to healthy cells [67, 68]. Therefore, careful selection of CAR-T antigens is critical to both enhance therapeutic efficacy and minimize off-target toxicity, ensuring the safety of the treatment.Moreover, adverse reactions related to CAR-T therapy, such as cytokine release syndrome (CRS) [69], hemophagocytic lymphohistiocytosis/macrophage activation syndrome (HLH/MAS) [70], and immune effector cell-associated neurotoxicity syndrome (ICANS) [71], remain significant clinical concerns. Following CAR-T reinfusion, activation, and expansion, macrophages release large amounts of cytokines, particularly IL-1 and IL-6, which contribute to CRS and ICANS. As solid tumors often present unique toxicities, further clinical trials and effective management strategies are needed to improve the safety and outcomes of CAR-T therapy.Recent advances in the use of single domain antibodies offer innovative solutions to some of these challenges. Single domain antibodies, with their small size and high specificity, may help in targeting tumor cells more precisely, thereby reducing off-target effects. Additionally, incorporating single domain antibodies into CAR-T cell designs could enhance targeting efficiency, potentially overcoming issues of antigen escape and minimizing toxicities related to off-target interactions.

### Characteristics of CAR-T therapy based on single domain antibody

**Fig. 3:**
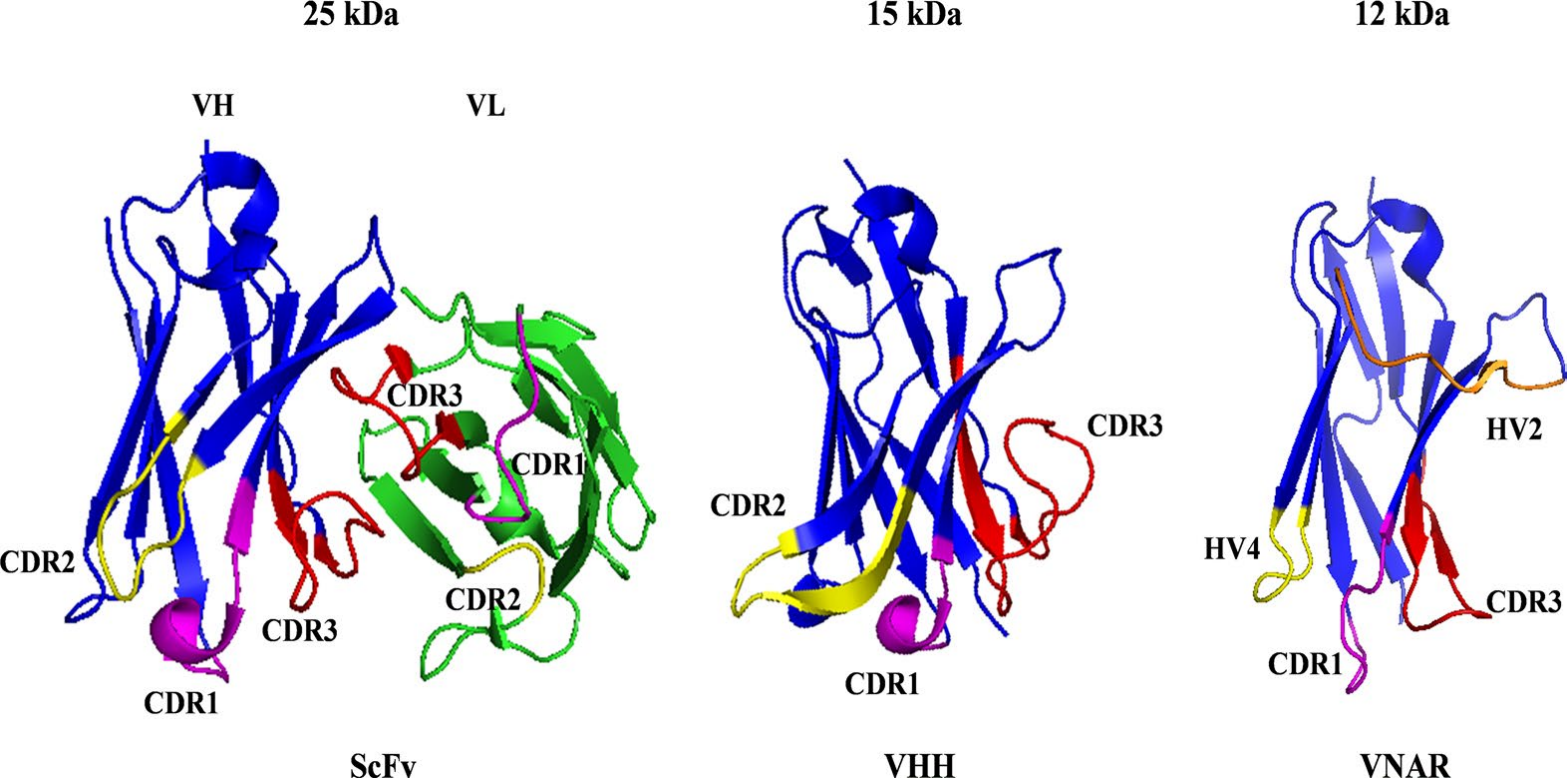
Structures of ScFv, VHH and VNAR with structural sequences derived from PDB: 7AQL, 1I3V and 8HT3. In ScFv and VHH, purple represents CDR1, yellow represents CDR2, and red represents CDR3. In VNAR, purple represents CDR1, gold represents HV2, yellow represents HV4, and red represents CDR3

In recent years, researchers have been focusing on other types of CAR domains, including single domain antibody, peptides, or ligands. Among them, single domain antibody as alternative CAR targeting domains show special advantages, including low immunogenicity, stability, specificity, and high affinity of single domain antibody, as well as a simple and feasible development process [71]. Many research results confirm that single domain antibody-based CAR-T can perform the same functions as single-chain antibody-based CAR-Ts in preclinical and clinical settings [72]. single domain antibody are heavy chain antibody variable regions that naturally lack light chains derived from camelids or sharks. The molecular weights of VHH and VNAR are only 15 kDa and 12 kDa, which are 1/10 of the molecular weight of conventional antibodies [14]. Their protein crystal structure measures 4 nm in length and 2.5 nm in diameter, earning them the name “nanobody”(Fig. 3).

Compared to ScFv, camel-derived VHH antibodies have four hydrophobic residues replaced by hydrophilic amino acids such as Phe37, Glu44, Arg45, and Gly47 [15], while shark-derived VNAR antibodies are rich in charged and hydrophilic amino acids, including conserved residues such as Glu46, Lys82, and Lys104 [73]. These residues form a charged structure through hydrogen bonding with each other and water molecules, creating a hydrophilic interface that avoids exposing larger hydrophobic regions to the solvent [73]. As a result, single domain antibody-based CARs exhibit minimal risk of aggregation. Additionally, the VHH sequence shares a high degree of similarity with human VH (VH3 gene family), reaching 75–90% similarity [74]. This similarity allows for easy humanization of the VHH gene, requiring only a few modifications to achieve human antibody characteristics, resulting in a low risk of immunogenicity [75].

VHH antibodies lack a light chain, which has led to the evolution of a longer CDR3 region (16–18 amino acids), enabling access to antigen-binding epitopes that IgG antibodies cannot reach. In sharks, the variable region domain consists of four hypervariable loops: CDR1, HV2, HV4, and CDR3, while the absence of CDR2 is due to somatic mutations in sharks, replaced by shorter hypervariable loops. Notably, the HV4 loop, located between HV2 and CDR3, is believed to play a crucial role in antigen binding [16]. Single domain antibodies also exhibit superior stability, including resistance to high temperatures, organic solvents, proteases, and a wider pH range (from pH 2 to 11), unlike traditional antibodies which are stable only within the pH range of 6 to 9 [76]. Furthermore, the preparation of single domain antibodies via engineered bacterial expression offers several advantages over the hybridoma cell method used for traditional antibodies, including ease of expression and genetic engineering. When constructing multivalent or multifunctional CARs, single domain antibodies can be efficiently linked through a simple linker to create multi-specific CARs.

**Fig. 4:**
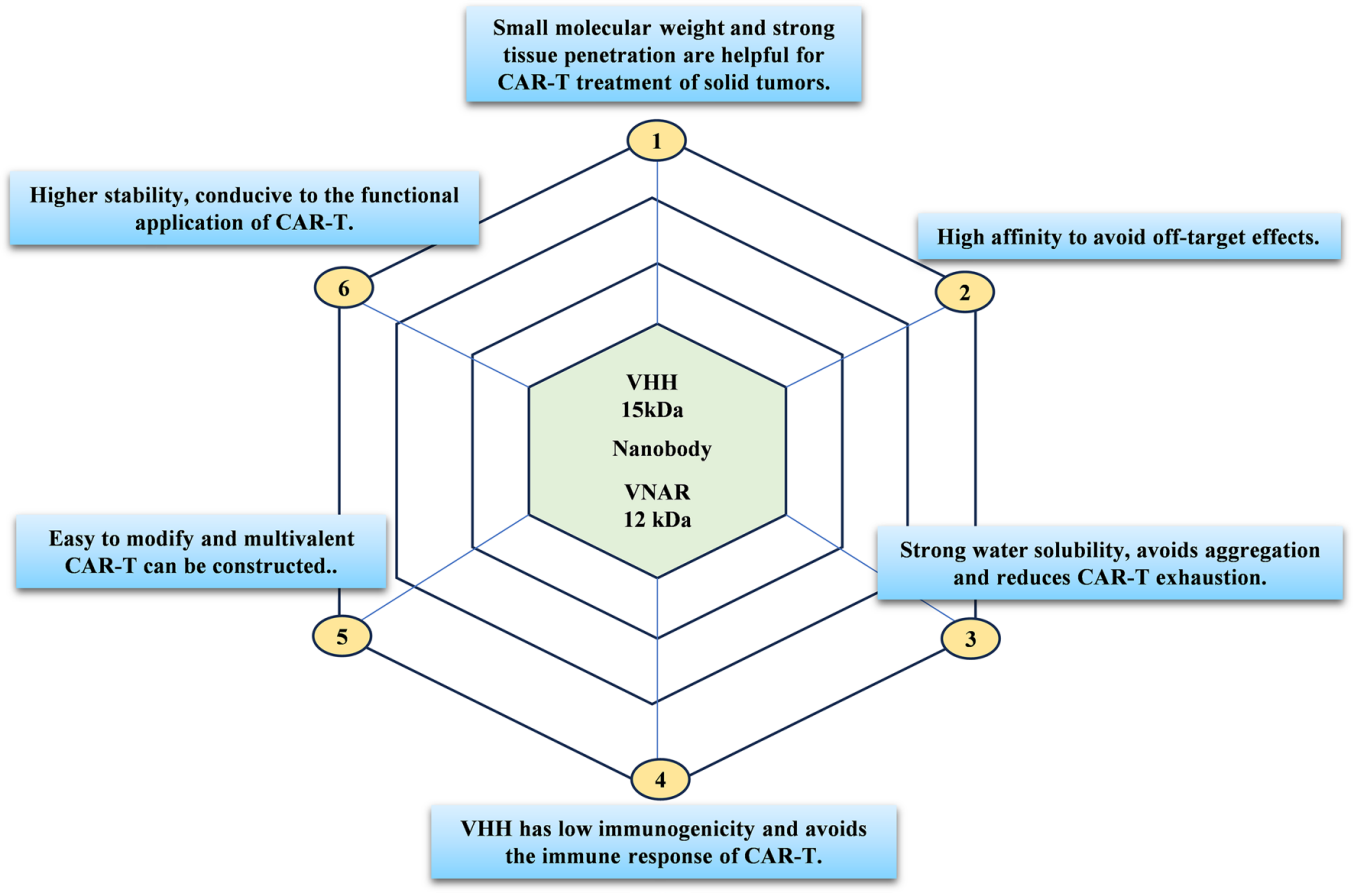
Advantages of single domain antibody for CAR-T therapy. VHH and VNAR have the characteristics of small molecular weight, strong tissue permeability, high affinity, good water solubility and difficulty in aggregation, low immunogenicity, and easy genetic modification, which are helpful for the further development of CAR-T

We summarize the key advantages of single domain antibodies for CAR-T therapy in Fig. 4, providing a more intuitive understanding of their unique characteristics.

### Target selection for single domain antibody-based CAR-T therapy

**Fig. 5:**
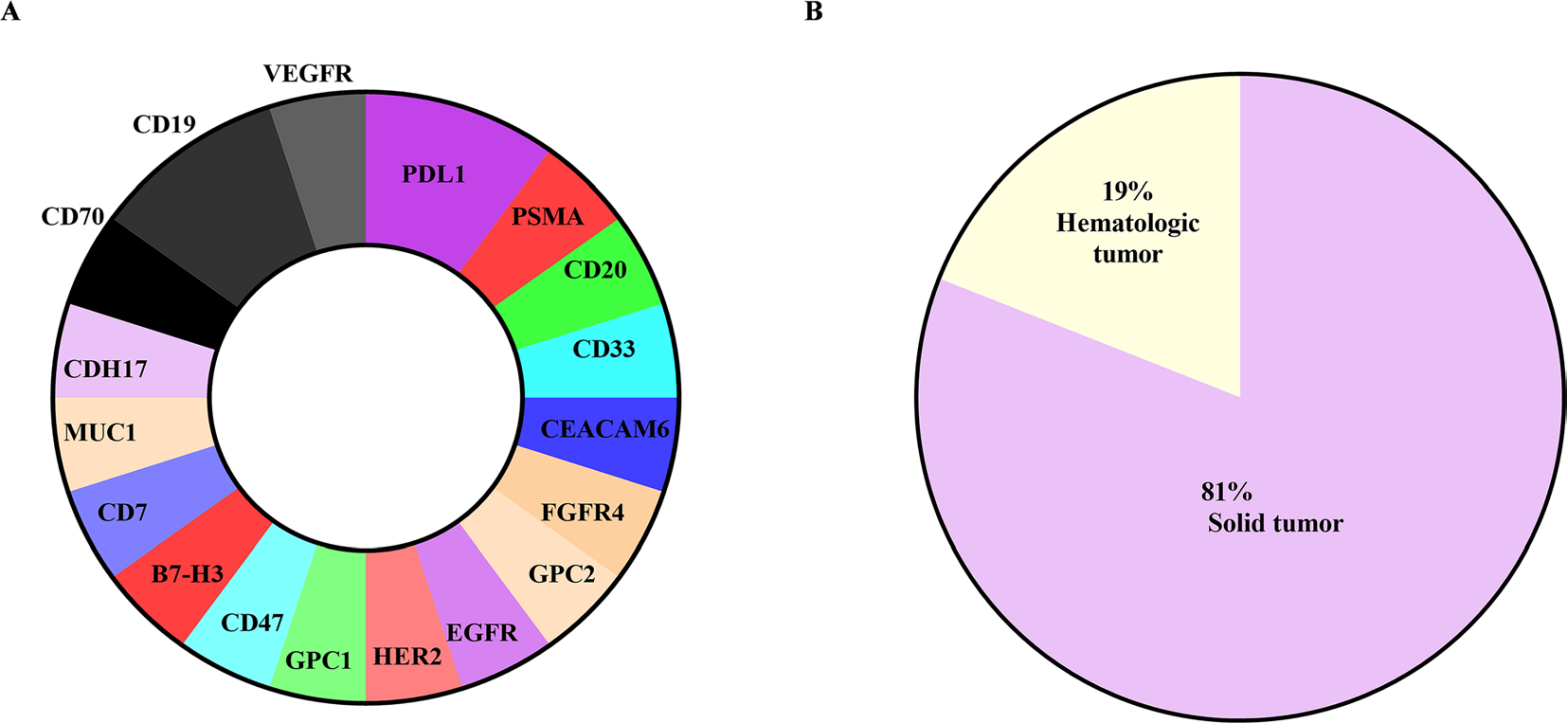
Selection of tumor types and targets treated with single domain antibody-based CAR-T therapy. (**A**). The therapeutic targets that have been used in the development of single domain antibody CAR-T therapy. The colored parts are targets related to solid tumors, and the brunet are targets related to blood tumors. (**B**). Statistics on the treatment proportion of single domain antibody CAR-T therapy in solid tumors and hematological tumors

According to several literature reports, CAR-T research based on single domain antibody targeting domains involves multiple popular targets. We summarize the targets and tumor types treated by single domain antibody CAR-T in the literature in Fig. 5. We can find that in single domain antibody-based CAR-T cell therapy, the selected target is no longer just the CD19 target that is common in CAR-T treatment (brunet part in Fig. 5A). Different types of targets are selected and applied, including HER2, PDL1, and EGFR. Popular targets such as CDH17, B7-H3, and FGFR4 are also less studied targets (colored part in Fig. 5A), and they are all used to treat solid tumors (Fig. 5B), which shows that single domain antibody could be used to treat solid tumors. CAR-T cells targeting the binding domain are effective against solid tumors and have great potential for development.

At present, most of the CAR-T target antigens are extracellular proteins. We further summarize the antibody types, targets, and tumor types currently used in single domain antibody-based CAR-T cells in Table 2. Then, we will briefly introduce the characteristics of popular CAR-T target antigens.

#### CEACAM

Carcinoembryonic Antigen-Related Cell Adhesion Molecule (CEACAM) is a family of glycoproteins involved in cell adhesion, immune regulation, and cancer progression [77]. The most well-known members include CEACAM1, CEACAM5 (also called carcinoembryonic antigen or CEA), and CEACAM6. CEACAM5 and CEACAM6 have emerged as promising targets for CAR-T therapy, particularly in colorectal and pancreatic cancers. Researchers, including I. Jancewicz et al., developed a novel CEACAM-targeted 2A3 single domain antibody chimeric antigen receptor (CAR-T) cell. The study showed that 2A3-CAR T cells exhibited strong activation and high cytotoxicity against CEACAM5 and/or CEACAM6 high-expressing cell lines, suggesting significant cross-reactivity. In vitro and in vivo assessments provided strong evidence for the potential of 2A3-CAR T cells in treating CEACAM5 and CEACAM6 overexpressing tumors [78].

#### MUC1

MUC1, a heterodimeric surface glycoprotein, is abnormally overexpressed in several cancers, particularly breast cancer, due to gene modifications and transcriptional dysregulation. Single domain antibody-based MUC1-redirecting CAR-T has shown promise as an effective and low-immunogenic cancer immunotherapy approach. This strategy effectively targets MUC1-overexpressing tumor cells, leading to efficient anti-tumor responses while minimizing off-target effects [79].

#### VEGFR2

VEGFR2 is a receptor often overexpressed in cancers such as head and neck squamous cell carcinoma. As a result, it has become a target for CAR-T therapy. In recent studies, single domain antibody-based VEGFR2-redirecting CAR-T cells were activated and expressed CD69 and CD25 upon co-culture with VEGFR2-expressing target cells. These CAR-T cells also demonstrated significant reliance on the secretion of cytokines like IL-2 and IFN-γ. Research by Fatemeh Hajari Taheri et al. demonstrated that VHH-based CAR T cells showed strong proliferation, cytokine secretion, and specific cytotoxicity against VEGFR2-expressing cells [81].

#### HER2

HER2, a member of the epidermal growth factor receptor family, is overexpressed in various tumors, including breast and gastric cancer. Single domain antibody-based HER2-redirecting CAR-T has been shown to enhance CAR-T cell amplification, cytokine secretion, and anti-tumor activity [82]. By combining oligoclonal single domain antibodies with third-generation CARs, significant improvements in tumoricidal activity and targeting specificity have been achieved, demonstrating the potential for better treatment outcomes in HER2-positive cancers [83].

#### PSMA

Prostate-specific membrane antigen (PSMA) is widely expressed in prostate tissue and is a key diagnostic and therapeutic target for prostate cancer. Single domain antibody-based PSMA-redirecting CAR-T has been developed to target PSMA-expressing tumor cells. These CAR-T cells showed activation marker expression (CD69) upon co-culture with PSMA-positive cells, as well as antigen-specific amplification and cytokine secretion, demonstrating the potential for targeted therapy in prostate cancer [84].

#### GPC2

GPC2 (Glypican-2) is a transmembrane heparan sulfate proteoglycan that plays a role in neuronal cell adhesion. VHH-based GPC2-redirecting CAR-T cells have been shown to inhibit active β-catenin signaling by disrupting the interaction between GPC2 and Wnt3a. These CAR-T cells demonstrated potent anti-tumor activity against neuroblastoma cells that express high levels of GPC2, making it a promising target for neuroblastoma therapy [85].

#### PD-L1

The PD-L1 checkpoint inhibitor is a well-known target in cancer immunotherapy. PD-L1-targeting CAR-T cells can relieve immune suppression in the tumor microenvironment while allowing CAR-T cells to activate against tumor cells [86]. Y. J. Xie et al. demonstrated that CAR-T cells targeting PD-L1 could reduce tumor growth and improve survival outcomes in animal models, suggesting the potential for reprogramming the tumor microenvironment and enhancing CAR-T therapy in solid tumors [87].

**Table 2:** The applications of single domain antibody in CAR-T therapies

Target	Type	Applicable treatment	Ref.
CD7	Alpaca VHH	Lymphocytoma.	[88]
CDH17	Alpaca VHH	Neuroendocrine tumors and gastrointestinal tumors	[89]
PDL1	Shark VNAR	Melanoma	[90]
PDL1	Alpaca VHH	Breast and liver cancer	[91]
VEGFR2	Alpaca VHH	Hemangioma	[80]
CD20/CD33	Alpaca VHH	Hepatobiliary carcinoma	[91]
CECAM6	Alpaca VHH	Pancreatic cancer	[78]
FGFR4	Alpaca VHH	Hepatocellular carcinoma	[92]
CD22	Alpaca VHH	Lymphocytoma.	[93]
CD19	Alpaca VHH	Lymphocytoma.	[94]
CD70	Alpaca VHH	Renal cell carcinoma	[95]
GPC1	Alpaca VHH	Pancreatic cancer	[96]
GPC2	Alpaca VHH	Neuroblastoma	[85]
PSMA	Alpaca VHH	Prostate cancer	[84]
B7-H3	Alpaca VHH	Pancreatic cancer	[97]
CD70	Alpaca VHH	Acute myeloid leukemia	[98]
CD47	Alpaca VHH	Melanoma	[99]
EGFR	Alpaca VHH	Human epithelial cell carcinoma.	[100]
HER2	Alpaca VHH	Breast cancer	[83]
CD39	Alpaca VHH	Ovarian cancer	[101]

Intracellular antigens make up approximately 90% of tumor-associated antigens, yet al.most all FDA-approved and marketed cancer therapies target extracellular antigens. This limitation has spurred the development of new therapeutic approaches, such as TCR-like antibody CAR-T therapy, which focuses on intracellular antigens and significantly expands the potential target range of monoclonal antibodies. By targeting intracellular antigens, this therapy opens up the possibility of treating a variety of previously underexplored tumor antigens [102]. Intracellular antigens are generated inside the tumor cells, degraded by the proteasome, and presented on the cell surface as MHC-I peptide complexes. These complexes are crucial for immune surveillance, and antibodies designed to recognize MHC or peptide complexes—referred to as TCR-like or mimetic antibodies—can effectively mimic T-cell receptors. By binding to these complexes, TCR-like antibodies can activate T cells and induce their proliferation, triggering potent costimulatory signals to mount an immune response against the tumor [103–105].

While previous studies have utilized human and mouse ScFv antibodies to target intracellular antigens, several challenges remain, particularly in terms of enhancing antigen-binding specificity and improving production efficiency. In the context of TCR-like antibody CAR-T therapy, there is an urgent need to refine these factors to achieve optimal therapeutic outcomes. Single-domain antibodies present a promising alternative to traditional ScFv antibodies, given their inherent advantages such as smaller size, higher stability, faster production, and the potential for improved specificity [102]. Single-domain antibodies, such as VHHs or VNARs, are smaller and more stable than conventional antibodies, making them easier and cheaper to produce. These antibodies also offer greater flexibility in terms of antigen binding, with the ability to recognize epitopes that traditional antibodies may miss. This makes them an attractive option for targeting intracellular antigens in CAR-T therapies. However, the application of single-domain antibodies in TCR-like CAR-T therapy remains an exciting area for further exploration, as their efficacy in targeting MHC/peptide complexes for intracellular targets is still under investigation.

## Structureal advantages of single domain antibody-based CAR-T

### The composition and structure of CAR-T cells: advances and innovations

**Fig. 6:**
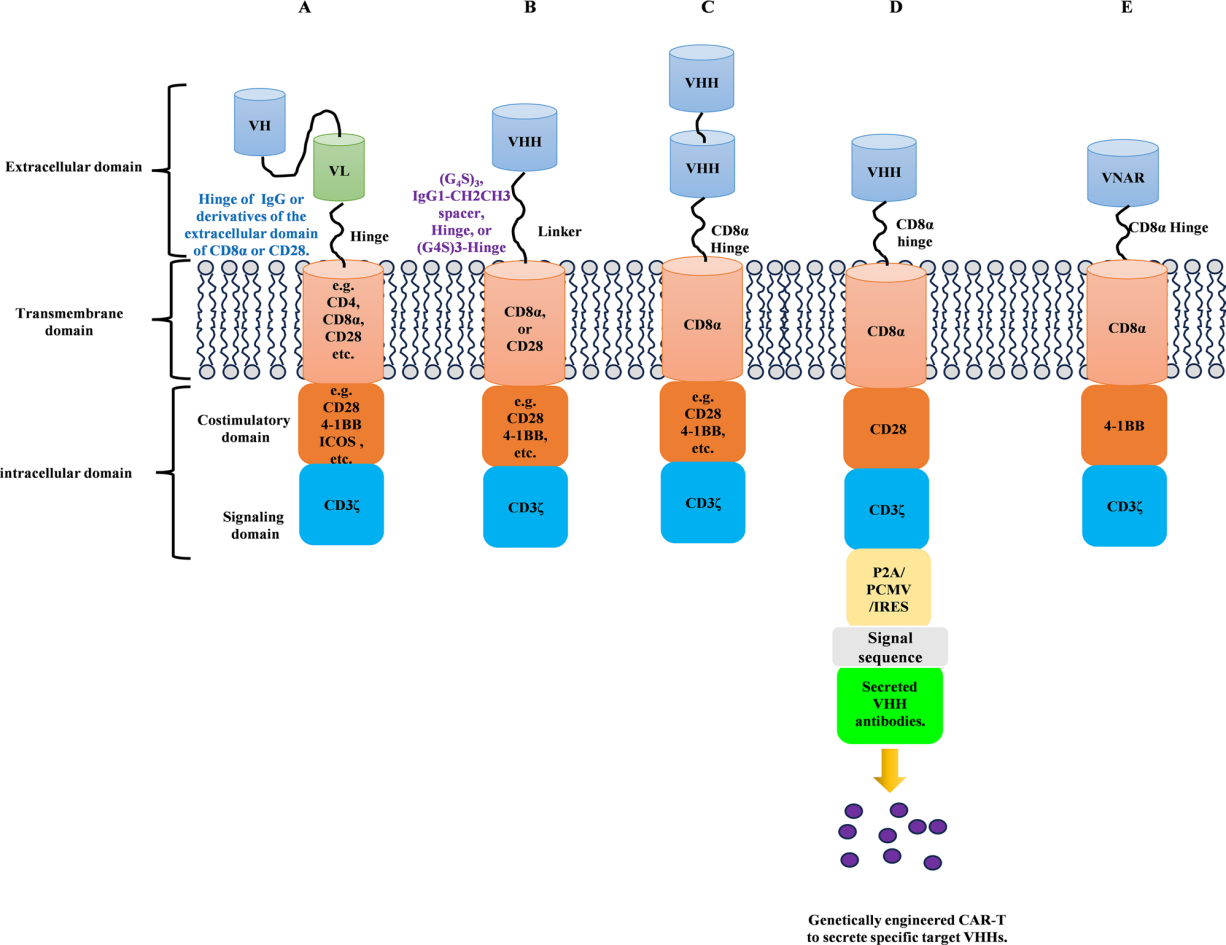
Single domain antibody-based CAR-T cell structure. (**A**). CAR-T cell structure of traditional ScFv. (**B**). CAR-T cell structure of monovalent VHH antibodies. (**C**). CAR-T cell structure of bivalent VHH antibodies. (**D**). CAR-T cell structure secreting VHH antibodies. E. Shark VNAR-based CAR-T cell structure

Chimeric Antigen Receptor T-cell (CAR-T) therapy has revolutionized cancer immunotherapy by enabling T cells to target specific tumor antigens. CARs are primarily composed of four main components (Fig. 6A):

1. Extracellular Antigen Recognition Domains: Most commonly, single-chain antibody fragments (ScFvs) are used to recognize specific tumor antigens.
2. Structural Components: These include hinges and transmembrane domains, providing stability and membrane integration for the CAR.
3. Costimulatory Signaling Domains: These domains, such as CD28 and 4-1BB, are essential for enhancing CAR-T cell activation, proliferation, and sustained effector function.
4. Activation Domain: The CD3ζ domain is crucial for T-cell activation, mediating downstream signaling to trigger immune responses [106].

Current CAR-T constructs also incorporate two costimulatory molecules, improving the efficacy of T-cell activation while overcoming immune tolerance. Genetic modifications further enable CAR-T cells to secrete cytokines, enhancing their therapeutic effect, particularly in the treatment of solid tumors. The ability to deliver specific cytokines at the tumor site is believed to alter the tumor microenvironment (TME) in a way that boosts CAR-T cell efficacy [107].

### VHH nanobodies in CAR-T therapy

In recent years, the use of single-domain antibodies, such as VHHs (derived from alpacas), has gained significant attention in CAR-T therapy. VHH nanobodies are small, highly stable, and capable of binding antigens with high specificity. Compared to traditional antibodies, VHHs offer distinct advantages, including a smaller molecular size, enhanced stability, and easier production, making them particularly suitable for CAR construction.

De Munter S et al. enhanced the antitumor activity of CD70-targeting VHH CAR-T cells and effectively reduced antigen-induced exhaustion by knocking out the CD70 gene. This genetic modification significantly improved the persistence and functionality of VHH CAR-T cells, highlighting the critical role of nanobodies in overcoming CAR-T cell exhaustion [108].Furthermore, Zhu Z et al. developed a CD5-targeting VHH CAR-γδT cell using mRNA engineering for the treatment of T-cell acute lymphoblastic leukemia (T-ALL). The mRNA-based approach enabled transient CAR expression, effectively reducing long-term toxicity risks. Additionally, the high penetration and specificity of VHH nanobodies further enhanced γδT cell-mediated cytotoxicity against T-ALL [109].

Structurally, the intracellular and transmembrane regions of VHH-based CARs are similar to traditional CARs. For example, the transmembrane domain typically includes CD8α and CD28, while the intracellular costimulatory domains primarily involve CD28 and 4-1BB, with CD3ζ as the activation domain [99]. The key difference lies in the spacer region between the antibody domain and transmembrane region. Traditional CARs usually incorporate IgG-derived hinge regions, whereas VHH-based CARs often utilize (G_4_S)_₃_ flexible linkers or a combination of these linkers with IgG or CD8α ectodomains (Fig. 6B). Moreover, they found that the secrected anti-CD47 VHH could bind to any existing cells, regardless of whether these cells had been transduced [99]. Meanwhile, treatment with CAR secreting anti-CD47 VHH further delayed the growth of syngeneic tumors (Fig. 6D). The optimal spacer length is crucial, as it ensures the proper spatial positioning of the CAR-T cell, facilitating efficient immune synapse formation and ultimately enhancing CAR-T cell activation and antitumor efficacy.

### TanCARs and multi-domain CAR-T cells

Another promising advancement is the development of Tandem CARs (TanCARs), which aim to overcome the limitations of ScFvs by using bispecific antigen-binding domains. TanCARs target two different epitopes of the same antigen or two distinct antigens on different tumor cells, offering improved tumor targeting and enhanced therapeutic efficacy (Fig. 6C). For example, F. Nie et al. developed bivalent VHH CAR-T cells that target dual epitopes of D3LL and a novel multi-chain CAR using the TREM1 receptor and DAP12, which significantly enhanced cytotoxic activity against small cell lung cancer [110]. Wu S et al. developed a humanized CD19/BAFFR bicistronic CAR-T cell using VHH as the antigen recognition domain, which significantly enhanced antitumor activity against B-cell malignancies. The dual-targeting design (CD19 and BAFFR) effectively reduced the risk of tumor escape, while the high affinity and stability of VHH improved the specificity and persistence of CAR-T cells [111]. Additionally, Lu Q et al. engineered a universal VHH CAR-T cell targeting BCMA/CD47 for the treatment of multiple myeloma. This design (BCMA and CD47) enhanced CAR-T cell antitumor activity, reduced manufacturing costs, and increased clinical applicability [112].The creation of TanCARs demonstrates that by modifying the traditional CAR-T structure—especially the transmembrane and intracellular regions—stronger therapeutic effects can be achieved. This is particularly important for solid tumors, which often present challenges such as dense extracellular matrices and inhibitory checkpoint signals that hinder immune responses. Engineering CAR-T cells to secrete cytokines such as IL12, IL15, and IL18 can help modify the TME and promote T cell activation, overcoming some of these challenges.

### VNAR antibodies: a new frontier for CAR-T therapy

VNARs (Variable New Antigen Receptors), derived from sharks, are another promising class of single-domain antibodies with a unique structure that can be leveraged in CAR-T therapy. These antibodies are smaller and more stable than traditional antibodies, providing potential advantages in targeting difficult epitopes and tissues.For example, D. Li et al. constructed a semi-synthetic shark VNAR phage library and developed an anti-PD-L1 VNAR (B2) that cross-reacts with human, mouse, and canine PD-L1. The B2 VNAR partially blocks the interaction between PD-1 and PD-L1, and CAR(B2) T cells effectively targeted PD-L1-expressing cancer cells, preventing tumor metastasis [90]. This study highlights the potential of VNAR-based CAR structures in not only monovalent but also bispecific CAR-T cells, offering promising new avenues for immunotherapy.The development of VNAR-based CAR-T cells targeting immune checkpoints and tumor antigens holds significant promise for improving CAR-T cell therapies, especially in overcoming the immune evasion mechanisms employed by solid tumors.

### Clinical progress and challenges of VHH- and VNAR-based CAR-T therapy in cancer treatment

VHH- and VNAR-based CAR-T therapies have demonstrated significant potential in cancer treatment. Beyond CARVYKTI, several other drug candidates are currently in clinical development, detailing key clinical trial data, including target antigens, tumor types, clinical phases, efficacy outcomes, and limitations.To date, only two VHH based CAR-T therapies for solid tumors are in preclinical studies (as summarized in Table 3). Despite some progress, these therapies still face several major challenges [113–114]: The immunosuppressive tumor microenvironment (TME) restricts CAR-T cell infiltration and persistence; heterogeneous antigen expression leads to inconsistent therapeutic efficacy; cytokine release syndrome (CRS) and on-target off-tumor toxicity remain significant safety concerns. In addition, VHH/VNAR constructs also have limitations, such as production challenges and immunogenicity issues, which should be considered to maintain a balanced perspective in the research.

Future research should focus on developing dual- or multi-target CAR-T therapies to address tumor heterogeneity, combining CAR-T therapy with immune checkpoint inhibitors (e.g., PD-1/PD-L1 inhibitors) or radiotherapy to enhance efficacy, and utilizing gene-editing technologies (such as CRISPR) to develop universal CAR-T cells that reduce manufacturing costs.

**Table 3:** The latest clinical research progress of CAR-T therapy based on VHH

Name	Target antigen	Clinical phase	Tumor type	Sponsor	Clinical trial registration number/ Patent publication numb	Limitations
LCAR-AIO	CD19 Molecule(CD19);CD20 Antigen (CD20);CD22 Molecule (CD22	Phase I clinical trial	Acute lymphocytic leukemia	Nanjing Duly Biotechnology Co., Ltd (Nanjing, China)	NCT06653556, NCT05318963, NCT05292898	CAR-T cell activation may trigger CRS.
SC-DARIC33	Sialic Acid-Binding Ig-Like Lectin 3(CD33)	Phase I clinical trial	Acute myeloid leukemia	Seattle Children’s Research Institute	NCT05105152	Bone marrow suppression and other side effects. Low CD33 expression in some AML patients may affect therapeutic efficacy.
anti-VEGFR-2 CAR T-cell therapy	Vascular Endothelial Growth Factor Receptor 2(VEGFR2)	Preclinical	Solid tumors	Helix BioPharma Corp	WO2018126317A1	off-target toxicity; high expression of TGF-β and PD-L1, may restrict CAR-T cell infiltration and persistence,
anti-CEACAM6 CAR T-cell therapy	Cluster Of Differentiation 66c(CEACAM6)	Preclinical	Pancreatic tumor	National Research Council of Canada	WO2018014122A1	off-target toxicity; CAR-T cell activation may trigger CRS.

## Conclusion and perspectives

Single-domain antibody-based CAR-T therapies, particularly those utilizing VHH and VNAR nanobodies, represent a transformative approach in cancer immunotherapy. Their small size, high stability, and superior tissue penetration make them uniquely suited to address the challenges of solid tumors, including the immunosuppressive tumor microenvironment (TME), tumor heterogeneity, and antigen accessibility. Innovations such as bispecific targeting, optimized spacer design, and the integration of shark-derived VNARs have further enhanced the precision and efficacy of these therapies. For instance, bispecific VHH-based CAR-T cells can simultaneously target multiple tumor antigens, reducing the risk of immune escape, while VNAR-based constructs have demonstrated exceptional affinity and specificity in preclinical models, particularly against challenging targets like PD-L1. Despite these advancements, several challenges remain. Targeting complex antigens such as O-GlcNAc glycosyltransferase (OGT) [115] and trophoblast surface antigen 2 (TROP2) [116] requires further optimization of nanobody design and CAR-T cell engineering. Additionally, the immunosuppressive nature of the TME and the potential for on-target/off-tumor toxicity necessitate continued innovation in CAR-T cell modulation and safety controls. Emerging technologies, such as artificial intelligence (AI)-driven target discovery and high-throughput screening, are poised to accelerate the identification of novel tumor-specific antigens and improve the precision of CAR-T therapies.

The future of nanobody-based CAR-T therapy lies in multidisciplinary collaboration, integrating advances in nanotechnology, immunology, and molecular biology to overcome current limitations. Regulatory support and adaptive policies will also be critical to facilitate the clinical translation of these therapies. As research progresses, VHH and VNAR-based CAR-T cells are expected to play a pivotal role in revolutionizing the treatment of solid tumors, offering new hope for patients with limited therapeutic options. By addressing the unique challenges of solid tumors and leveraging the unique properties of nanobodies, these therapies hold the potential to significantly improve patient outcomes and redefine the landscape of cancer immunotherapy.

## Data Availability

No datasets were generated or analysed during the current study.

## Abbreviations

CAR-T: Chimeric antigen receptor T cell
ScFv: Single-chain variable fragment
VH: Heavy chain variable
VL: Light chain variable
VHH: Variable domain of heavy chain of HCAb
VNAR: Variable New Antigen Receptor
HER2: Human epidermal growth factor receptor 2
PDL1: Programmed cell death 1 ligand 1
EGFR: Epidermal growth factor receptor
VEGFR: Vascular Endothelial Growth Factor Receptor
CECAM: Human carcinoembryonic antigen related cell adhesion
GPC: Glypican
PSMA: Prostate specific membrane antigen
BCMA: B-cell maturation antigen
MUC1: Mucl protein
FGFR: Fibroblast growth factor receptor
OGT: O-GlcNAc transferase
TROP2: Trophoblast cell surface antigen 2
TME: Tumor microenvironment
CRS: Cytokine release syndrome
HLH: Hemophagocytic syndrome
MAS: Macrophage activation syndrome
ICANS: Immune effector cell-associated neurotoxicity syndrome
